# Heterotopic Pancreas Leading to Ileo-Ileal Intussusception

**Published:** 2012-06-01

**Authors:** KN Ratan, Mahavir Singh, Babita Rani, Tina

**Affiliations:** Departments of Pediatric Surgery, Pt. BD Sharma PGIMS Rohtak, Haryana, India.; Departments of Pediatric Surgery, Pt. BD Sharma PGIMS Rohtak, Haryana, India.; Departments of Community Medicine, Pt. BD Sharma PGIMS Rohtak, Haryana, India.; Departments of Anaesthesiology, Pt. BD Sharma PGIMS Rohtak, Haryana, India.

**Keywords:** Intussusception, Heterotopic pancreas, Lead point

## Abstract

A heterotopic pancreas as the lead point of ileo-ileal intussusception is extremely rare. A 12-year-old previously healthy boy, presented to the emergency room with the complaint of severe abdominal pain for the last 6-8 hours. A preoperative diagnosis of ileo-ileal intussusception was made on ultrasound and an emergency exploratory laparotomy was done. At laparotomy an ileo-ileal intussusception was found and a polyp noted as a lead point. On histopathology this polyp was found to be heterotopic pancreas.

## INTRODUCTION

A heterotopic pancreas (HP), a developmental anomaly, is defined as pancreatic tissue found on ectopic sites without contiguity with the main pancreas [1]. The presence of heterotopic pancreas is unusual with an estimated incidence of 0.2% of upper abdominal operations [2]. HP occurs predominantly in the stomach, duodenum and proximal jejunum. A heterotopic pancreas of the ileum is rare and usually found in a Meckel’s diverticulum, which may cause intussusception in childhood. An isolated heterotopic pancreas as the lead point of intussusception is extremely rare especially in children. Even after extensive literature search we could retrieve only 5 cases of isolated heterotopic pancreas as the lead point of intussusception [2-6]. We report a case of heterotopic pancreas of ileum presenting as ileo-ileal intussusception.

## CASE REPORT

A 12-year-old boy presented in paediatric emergency room with complaints of severe paroxysmal colicky abdominal pain for the last 6-8 hours associated with non-bilious vomiting. Patient was apprehensive and looked pale. Patient had passed stool in the morning with no history of blood or mucous in the stool. On per abdominal examination no lump was palpable. There were no signs of peritonitis. His laboratory investigations were within normal limits. Abdominal radiograph showed air fluid levels indicative of a small-bowel obstruction. Ultrasonography revealed ileo-ileal intussusception. After resuscitation, patient underwent emergency laparotomy. At operation an ileo-ileal intussusception was found (Fig. 1). The invaginated segment was situated approximately 40 cm from the ileo-cecal valve. The reduction of intussusception was carried out gently. After reduction the adjacent small intestine had a normal color and peristalsis. A firm polypoid mass was palpable in the lumen of ileum about 20 cm from ileo-cecal valve (Fig. 2,3). An enterotomy confirmed the presence of a polypoid lesion arising from antimesenteric border. This segment of ileum was resected and an end-to-end anastomosis performed. Postoperative recovery was uneventful. Histopathological examination revealed that the mass was composed of mature pancreatic acini and ducts.

**Figure:**
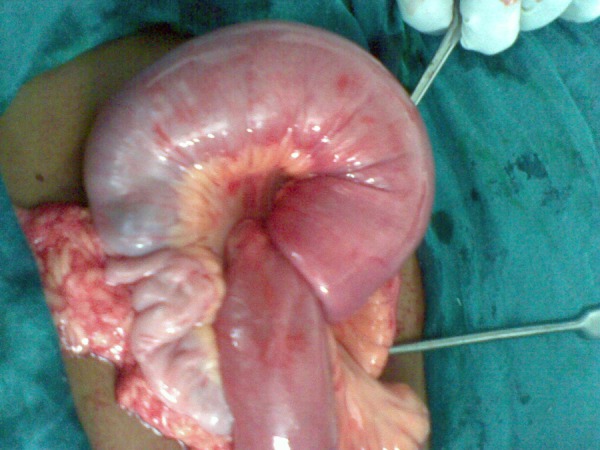
Figure 1: Ileo-ileal intussusception.

**Figure:**
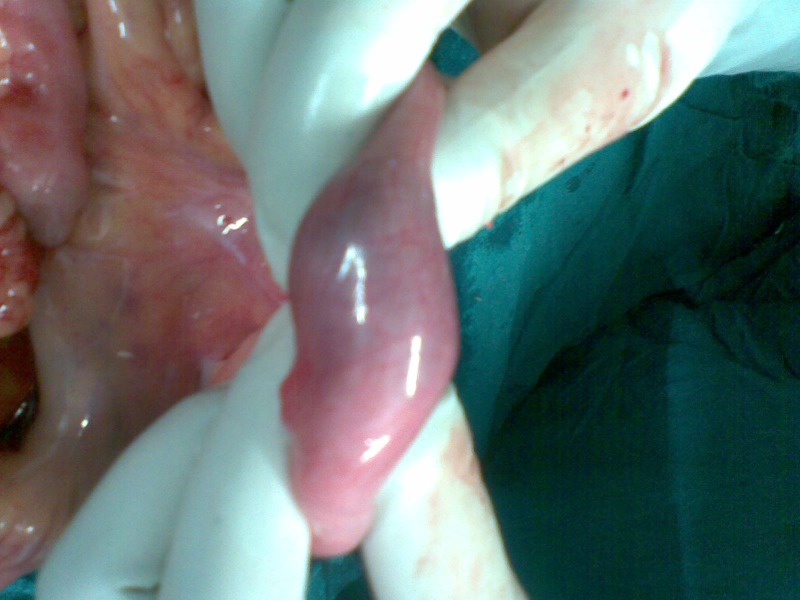
Figure 2: Lead point being palpated.

**Figure:**
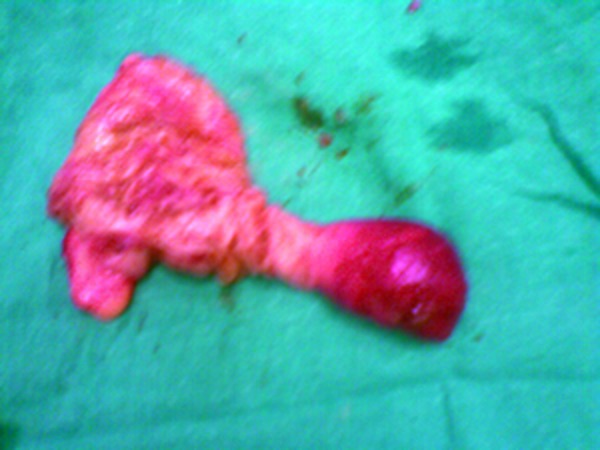
Figure 3: Polyp-lead point.

## DISCUSSION

Intussusception is the most common cause of intestinal obstruction in children under 5 years of age with most of the cases being reported between 6 and 18 months of age. In most cases, the intussusceptions are idiopathic. Pathological lead points (PLP) contribute to 2–10% of all intussusceptions. Common pathological lead points are Meckel’s diverticulum, appendix, hamartomas, lipomas, leiomyomas, neurofibromas, adenomas, various types of polyps, parasitic infestation and adhesions. The incidence of lead points increases with age and in children over 4 years of age; 57% of intussusceptions have lead points [7].

Heterotopic pancreas is a rare PLP. It has been suggested that heterotopic pancreas results from the separation of pancreatic tissue during the embryonic rotation of the dorsal and ventral buds [8]. The less common sites of HP include the esophagus, lungs, gallbladder, spleen, umbilicus, fallopian tubes, lymph nodes, mediastinum, tongue and submandibular salivary gland [9]. Most heterotopic pancreas cases are asymptomatic and discovered incidentally during surgery or autopsy. The lesion in the ileum is almost always asymptomatic and seldom causes intussusception. Intussusception caused by heterotopic pancreas is rare but has been described previously. Most series that have described this complication noted heterotopic pancreas to be located within the ileum where the concomitant existence of a Meckel’s diverticulum is thought to exacerbate the ability of the heterotopic pancreatic tissue as a lead point for the intussusception. The isolated heterotopic pancreas of the ileum with no Meckel’s diverticulum causing intussusception as reported here is extremely rare. 

## Footnotes

**Source of Support:** Nil

**Conflict of Interest:** None declared
